# Promoting resilience in stress management (PRISM) for patients with early stage breast cancer: a pilot feasibility study

**DOI:** 10.1007/s10549-025-07741-3

**Published:** 2025-06-16

**Authors:** Gabrielle B Rocque, Etzael Ortiz Olguin, Jessi M. Shuey, Tanvi Padalkar, Courtney P. Williams, Andres Azuero, Ana Falcao, Nicole L. Henderson, Chloe J. Taub, Molly Ream, Joyce P. Yi-Frazier, Courtney C. Junkins, Katherine Reeder-Hayes, Abby R. Rosenberg

**Affiliations:** 1 Department of Medicine, Division of Hematology Oncology, University of Alabama at Birmingham, 1808 7 Th Avenue S, Birmingham, AL 35233 USA; 2 O’Neal Comprehensive Cancer Center, University of Alabama at Birmingham, Birmingham, AL USA; 3 Department of Medicine, Division of General Internal Medicine and Population Science, University of Alabama at Birmingham, Birmingham, AL USA; 4 School of Nursing, University of Alabama at Birmingham, Birmingham, AL USA; 5 Department of Supportive Oncology, Dana-Farber Cancer Institute, Boston, MA USA; 6 Department of Medicine, Division of Oncology, University of North Carolina at Chapel Hill, Chapel Hill, NC USA; 7 UNC-Lineberger Comprehensive Cancer Center, University of North Carolina at Chapel Hill, Chapel Hill, NC USA; 8 Department of Pediatrics, Boston Children’s Hospital, Boston, MA USA; 9 Department of Pediatrics, Harvard Medical School, Boston, MA USA

**Keywords:** Breast cancer, Pilot study, Resilience, Feasibility

## Abstract

**Purpose:**

Women with breast cancer often experience persistent psychological distress. Promoting resilience in stress management (PRISM) is a manualized, skills-based, psychosocial intervention shown to promote resilience and alleviate psychological distress among adolescents and young adults with cancer.

**Methods:**

This pilot, convergent mixed methods study examined PRISM’s feasibility and in-sample preliminary impact (single-group) on psychosocial outcomes of women with early stage breast cancer (EBC). Women receiving chemotherapy for stage I–III breast cancer completed six standard PRISM sessions focused on rapport building, stress management, goal setting, cognitive reframing, meaning-making, and family integration. Feasibility, the primary outcome, was defined as 70% of participants completing all intervention sessions and pre-post survey. Secondary outcomes included intervention acceptability, appropriateness, and 8 psychosocial outcomes. Patient perspectives on PRISM were elucidated via qualitative interviews and deductively analyze. Pre- and post-intervention changes in survey scores were analyzed using paired t-tests and Cohen’s *d* effect size.

**Results:**

Of 57 patients approached, 30 (53%) participated in PRISM; participants were 57% Black with median age of 51 years (IQR 47–59). PRISM sessions were feasible based on the 83% completion rate. Additional secondary implementation outcomes also demonstrated feasibility, acceptability, and appropriateness using validated survey measured. The largest effects were observed in participants’ resilience (*d* = 0.6), growth (*d* = 0.5), and self-improvement (*d* = 0.5). Interviews supported both feasibility and impact of PRISM.

**Conclusion:**

The PRISM-EBC intervention was feasible, and pre-post changes suggest potential benefit, warranting further investigation in a future randomized controlled trial.

## Introduction

Women with breast cancer often experience persistent psychological distress [1, 2]. Women who are younger, mothers, live in under-resourced areas, have limited social support, or with intersectional identities are at disproportionately higher risk of adverse psychological outcomes [1, 3]. For example, Black mothers have greater biologic markers of stress, such as allostatic load, as well as sociodemographic stressors such as poverty (20% Black vs. 8% White) and single parenthood (64% vs. 24%) than their White counterparts [4–6]. Furthermore, higher stress is associated with higher likelihood of breast cancer recurrence, higher fear of recurrence, and poorer quality of life during and after cancer therapy [7–10]. Thus, interventions are needed to reduce stress, particularly among marginalized populations at risk for disparate outcomes.

The Promoting Resilience In Stress Management (PRISM) intervention is an evidence-based program that builds resilience in persons living with serious illnesses, including cancer, by utilizing centrally administered, skills-based lay coaching to bolster positive psychological tools known as resilience resources, including stress management, goal setting, and positive reframing [11]. PRISM is delivered virtually, decreasing the burdens associated with accessing psychosocial support. This intervention was initially developed for adolescents and young adults (AYAs) with cancer and other serious illnesses, where it decreased psychological distress and improved resilience, hope, and quality of life [12–16]. Given its feasibility and acceptability in adult populations including adult patient populations, caregivers and healthcare providers [17, 18], PRISM is an attractive option to build resilience in adult women with early stage breast cancer. However, PRISM has not been tested for its effects on fear of recurrence or utilized in an adult cancer population. Therefore, we conducted an initial feasibility study aiming to test PRISM’s feasibility, acceptability, and appropriateness in women with early stage breast cancer. Additionally, we explored participant perspectives on how PRISM could meet the unique needs of women with breast cancer and assessed the change in patient-reported psychological outcomes for those receiving it.

## Methods

### Study design and sample

This mixed methods, feasibility study examined feasibility of the PRISM intervention on women with early stage breast cancer receiving chemotherapy at the University of Alabama at Birmingham (UAB) from February to September 2024. English-speaking women receiving chemotherapy at UAB for Stage I–III breast cancer were eligible for inclusion. The study coordinator approached eligible women in clinic and obtained informed consent. To assess feasibility, a sample size of 30 women with early stage breast cancer initiating the intervention was planned. This sample size was selected to provide a sufficient sample for qualitative analysis and effect size estimates. Purposive sampling was used to ensure that the 30 participants included a minimum of 10 who were Black, 10 living in rural areas, 5 mothers, and 5 individuals living in a disadvantaged area using the area deprivation index [19]. Participants received $100 for their participation. The UAB Institutional Review Board approved this study (IRB-300011028).

### PRISM intervention

PRISM is a manualized, skills-based intervention delivered by trained lay coaches targeting four resilience skills: stress management, goal setting, cognitive reframing, meaning-making, as well as an additional resource of family integration (Table 1) [12–16, 20].

**Table 1:** Promoting resilience in stress management (PRISM) intervention content

Session focus	Details	Format	Stress and coping theory constructs
1. Managing stress	Relaxation strategies (i.e., deep breathing), mindfulness techniques	1:1 (video)	Situational factors
2. Goal setting	Setting SMART goals, planning for roadblocks	1:1 (video)	
3. Positive reframing	Recognizing and reframing negative thoughts	1:1 (video)	Coping processes
4. Meaning making	Identifying gratitude or meaning from illness experience	1:1 (video)	
5. “Coming together”	Discussion about what was learned, what worked, how loved ones can help (optional)	Family meeting (video)	Situational factors and coping processes
6. Advance care planning	Discussion of advance care planning (optional)		
Skill practice	Between-session exercises to practice, further develop and track skills	Digital app	

Participants received 6, 30–60 min PRISM sessions every 1–2 weeks for 6–8 weeks. As in previous implementations of PRISM, sessions were delivered predominantly by videoconference with the option for phone or in-person sessions and sessions could be combined upon request. Participants also had access to a companion PRISM digital app to assist in practicing their learned skills between sessions. During the initial session, participants were instructed on how to download and navigate the app and reminded to utilize the app at subsequent sessions for practice. However, participants were not required to have a smartphone to participate. Participants completed questionnaire packets (described below) at baseline and within 60 days of PRISM completion.

### PRISM coach training

Details on coach training are described elsewhere [11–14]. In brief, the coach (EO) received over 8 h of standardized training and directed feedback on delivery of PRISM sessions. Coaching sessions were audio-recorded, and one of every five sessions was reviewed for fidelity by a trained clinical psychologist with expertise in the intervention. All sessions met fidelity benchmarks.

### Primary outcome

The primary outcome for this study was feasibility, defined as 70% of women who initiated the intervention completing all PRISM sessions and pre-post surveys.

### Secondary outcomes

*Quantitative:* Additional exploratory outcomes were captured using validated tools on the pre- and post-PRISM surveys. Acceptability, appropriateness, and feasibility of the intervention were measured on the post survey using the Acceptability of Intervention Measure (AIM), Intervention Appropriateness Measure (IAM), and Feasibility of Intervention Measure (FIM; scored 1–5, higher scores indicate better outcomes) [21]. Other outcomes captured included the Connor-Davidson Resilience scale (CD-RISC; scored 0–40, higher scores indicate greater resilience) [22], patient activation measure (PAM; scored 0–100, higher scores indicate greater activation) [23], post-traumatic growth inventory (PTGI; scored 0–105 with factor subscales ranging from 0–10 to 0–35; higher scores indicate more positive transformation) [24], fear of cancer recurrence inventory (FCRI-SF; scored 0–36, higher scores indicate greater fear of cancer recurrence) [25], patient health questionnaire (PHQ-8; scored 0–24, higher scores indicate worse depressive symptoms) [26], general anxiety disorder anxiety scale (GAD-7; scored 0–21, higher scores indicate worse anxiety [27], functional assessment of chronic illness therapy spiritual well-being tool (FACIT-Sp; scored 0–48 with each factor scored 0–16, higher scores indicate greater spiritual well-being) [28], and patient-reported outcomes measurement information system global health (PROMIS-Global; physical and mental health scored 4–20 with T-scores ranging from 16.2 to 67.7, higher T-scores indicate greater physical or mental health) [29]. An additional question assessed life stressors in addition to breast cancer in the previous 3 months (no vs. yes with free text).

Social risks and needs, including housing instability, food insecurity, transportation problems, utility help needs, financial strain, family and community support, and disability, were captured on the baseline survey using the Centers for Medicare and Medicaid Services’ Accountable Health Communities Health-Related Social Needs screening tool [30]. Sociodemographic factors [age, sex, race, address and zip code (to derive rurality [31] and area deprivation index) [19]] and cancer characteristics (subtype, stage, treatment) were abstracted from the electronic medical record.

*Qualitative:* After completing PRISM, all participants were invited to a 30- to 60-min semi-structured interview. The interview guide, developed by a medical anthropologist (NH) and oncologist (GR), elucidated perspectives about the intervention. A medical anthropologist (NH) conducted interviews in person or via telephone or videoconference. Interviews were audio-recorded and transcribed verbatim.

### Data analysis

*Quantitative:* Sociodemographic and outcome data were described using means and standard deviations (SD) or median and interquartile range (IQR) for continuous variables and frequencies and percentages for categorical variables. Pre- and post-intervention changes in survey scores were analyzed by computing effect sizes in the form of Cohen's d (standardized mean difference, using the pre-SD) and interpreted using Cohen’s guidelines [32] (small: *d* = 0.2, medium: *d* = 0.5, large: *d* = 0.8). Analyses were performed using SAS version 9.4 (SAS Institute, Cary, NC).

*Qualitative:* Interview data were managed and analyzed using Dedoose. An independent coder (TP) used the quantitative survey items to develop codes and deductively analyze qualitative data [33, 34]. Next, two coders (TP and AF) iteratively tagged excerpts of interviews that best represented key themes. This continued until a satisfactory interrater reliability was achieved (K > 0.7). Representative quotes were selected through consensus discussion within the broader research team, who also aided to refine and review any discrepancies in coding to ensure adequate representation of data and identify consistency in alignment with quantitative data.

*Mixed methods:* In this convergent design, quantitative and qualitative results were examined concurrently and integrated to provide a comprehensive understanding of the intervention’s feasibility and preliminary impact in this population [35]. The quantitative findings serve as the foundation, with elaboration through quotes from qualitative analysis.

## Results

### Study sample

From February to September 2024, 57 patients were approached; 32 (56%) consented to participate in the PRISM intervention, 30 (53%) initiated PRISM. Eligible patients (*n* = 25) declined participation due to not being stressed about diagnosis (*n* = 2), not interested (*n* = 15), too overwhelmed (*n* = 4), too sick (*n *= 1), declined treatment (*n* = 2), or already taking advantage of therapy sessions at UAB (*n* = 1). Two patients withdrew before starting coaching sessions. Of the 30 patients with early stage breast cancer participating, the median age was 51 years [IQR 47–59]. The majority of participants were Non-Hispanic Black (57%) and 37% were unemployed at the start of the study (Table 2). Nearly 3 out of 4 participants (73%) found it somewhat difficult to pay for basic necessities, 60% were food insecure, 43% had gone hungry within the previous year, 37% were at risk of having their utilities disconnected, and 27% were home insecure (Table 3).

**Table 2:** Participant sociodemographic characteristics

Variables	Started PRISM (*N* = 30)	Completed PRISM (*n* = 25)	Didn’t complete (*n* = 5)
Age (median (IQR))	51 years (47–59)	51 years (47–59)	48 years (44–57)
Sex (*n*, %)			
Female	30 (100)	25 (100)	5 (100)
Race/ethnicity (*n*, %)			
Non-Hispanic black	17 (57)	14 (56)	3 (60)
Non-Hispanic white	12 (40)	10 (40)	2 (40)
Hispanic	1 (3)	1 (4)	–
Education (*n*, %)			
High school, no degree	1 (3)	–	1 (20)
High school graduate/GED	4 (13)	3 (12)	1 (20)
Some college	7 (23)	5 (20)	2 (40)
Associate’s degree	5 (17)	5 (20)	–
Bachelor’s degree	4 (13)	3 (12)	1 (20)
Advanced degree	9 (30)	9 (36)	–
Marital status (*n*, %)			
Single, never married	4 (13)	1 (4)	3 (60)
Married/partnered	14 (47)	12 (48)	2 (40)
Separated/divorced	12 (40)	12 (48)	–
Employment status (*n*, %)			
Not currently employed	11 (37)	8 (32)	3 (60)
Part-time	6 (20)	6 (24)	–
Full-time	10 (33)	8 (32)	2 (40)
Retired	3 (10)	3 (12)	–
Insurance coverage (*n*, %)			
Private	19 (63)	16 (64)	3 (60)
Medicaid	7 (23)	5 (20)	2 (40)
Medicare	4 (13)	4 (16)	–
Rurality (*n*, %)			
Rural	1 (3)	–	1 (20)
Urban	29 (97)	25 (100)	4 (80)
Area deprivation index (*n*, %)			
Least disadvantaged (≤ 85)	22 (73)	19 (76)	3 (60)
Most disadvantaged (> 85)	7 (23)	5 (20)	2 (40)
Unknown	1 (3)	1 (4)	–
Current therapy			
Neoadjuvant	19 (63)	15 (60)	4 (80)
Adjuvant	11 (37)	10 (40)	1 (20)
Cancer stage (*n*, %)			
I	2 (7)	2 (8)	–
II	26 (87)	22 (88)	4 (80)
III	2 (7)	1 (4)	1 (4)
Cancer subtype (*n*, %)			
HR+HER2+	7 (23)	6 (24)	1 (20)
HR+HER2-	17 (57)	14 (56)	3 (60)
HR-HER2+	1 (3)	1 (4)	–
HR-HER2-	3 (10)	2 (8)	1 (20)
HR+HER2 unknown	1 (3)	1 (4)	–
HR-HER2 unknown	1 (3)	1 (4)	–

*HR ± * hormone receptor-positive/negative, *HER2* ± */unknown* human epidermal receptor 2-positive/negative/unknown

**Table 3:** Participant social determinants of health

Variable	Started PRISM (*N* = 30)	Completed PRISM (*n* = 25)	Didn’t Complete (*n* = 5)
Housing (*n*, %)			
Current living situation			
Steady living situation	22 (73)	20 (80)	2 (40)
Steady living situation, but worried about losing it in the future	7 (23)	4 (16)	3 (60)
Don’t have a steady living situation	1 (3)	1 (4)	–
Have problems with living situation (pests, mold, lead, leaks, etc.)?			
No	21 (70)	17 (68)	4 (80)
Yes	9 (30)	8 (32)	1 (20)
Food (*n*, %)			
Within the past 12 months, you worried that your food would run out before you got money to buy more			
Often true	5 (17)	4 (16)	1 (20)
Sometimes true	13 (43)	11 (44)	2 (40)
Never true	12 (40)	10 (40)	2 (40)
Within the past 12 months, the food you bought just did not last and you did not have money to buy more			
Often true	4 (13)	3 (12)	1 (20)
Sometimes true	9 (30)	9 (36)	–
Never true	17 (57)	13 (52)	4 (80)
Transportation (*n*, %)			
In the past 12 months, has lack of reliable transportation kept you from medical appointments, meetings, work, or from getting things needed for daily living?			
No	22 (73)	18 (72)	4 (80)
Yes	8 (27)	7 (28)	1 (20)
Utilities (*n*, %)			
In the past 12 months, has the electric, gas, oil, or water company threaten to shut off services in your home?			
No	19 (63)	17 (68)	2 (40)
Yes	11 (37)	8 (32)	3 (60)
Already shut off	–	–	–
Financial strain (*n*, %)			
How hard is it for you to pay for the very basics like food, housing, medical care, and heating?			
Not hard at all	8 (27)	7 (28)	1 (20)
Somewhat Hard	20 (67)	17 (68)	3 (60)
Very Hard	2 (7)	1 (4)	1 (20)
Family and community support (*n*, %)			
If for any reason you need help with day-to-day activities, do you get the help you need?			
No help needed	8 (27)	7 (28)	1 (20)
I get all the help I need	14 (47)	12 (48)	2 (40)
I need a little more help	8 (27)	6 (24)	2 (40)
I need a lot more help	–	–	–
How often do you feel lonely or isolated from those around you?			
Never	6 (20)	5 (20)	1 (20)
Rarely	11 (37)	8 (32)	3 (60)
Sometimes	9 (30)	9 (36)	–
Often	4 (13)	3 (12)	1 (20)
Always	–	–	–
Disabilities (*n*, %)			
Because of a physical, mental, or emotional condition, do you have serious difficulty concentrating, remembering, or making decisions?			
No	18 (60)	16 (64)	2 (40)
Yes	12 (40)	9 (36)	3 (60)
Because of a physical, mental, or emotional condition, do you have difficulty doing errands alone such as visiting a doctor’s office or shopping?			
No	23 (77)	18 (72)	5 (100)
Yes	7 (23)	7 (28)	–

### PRISM intervention feasibility

**Fig. 1:**
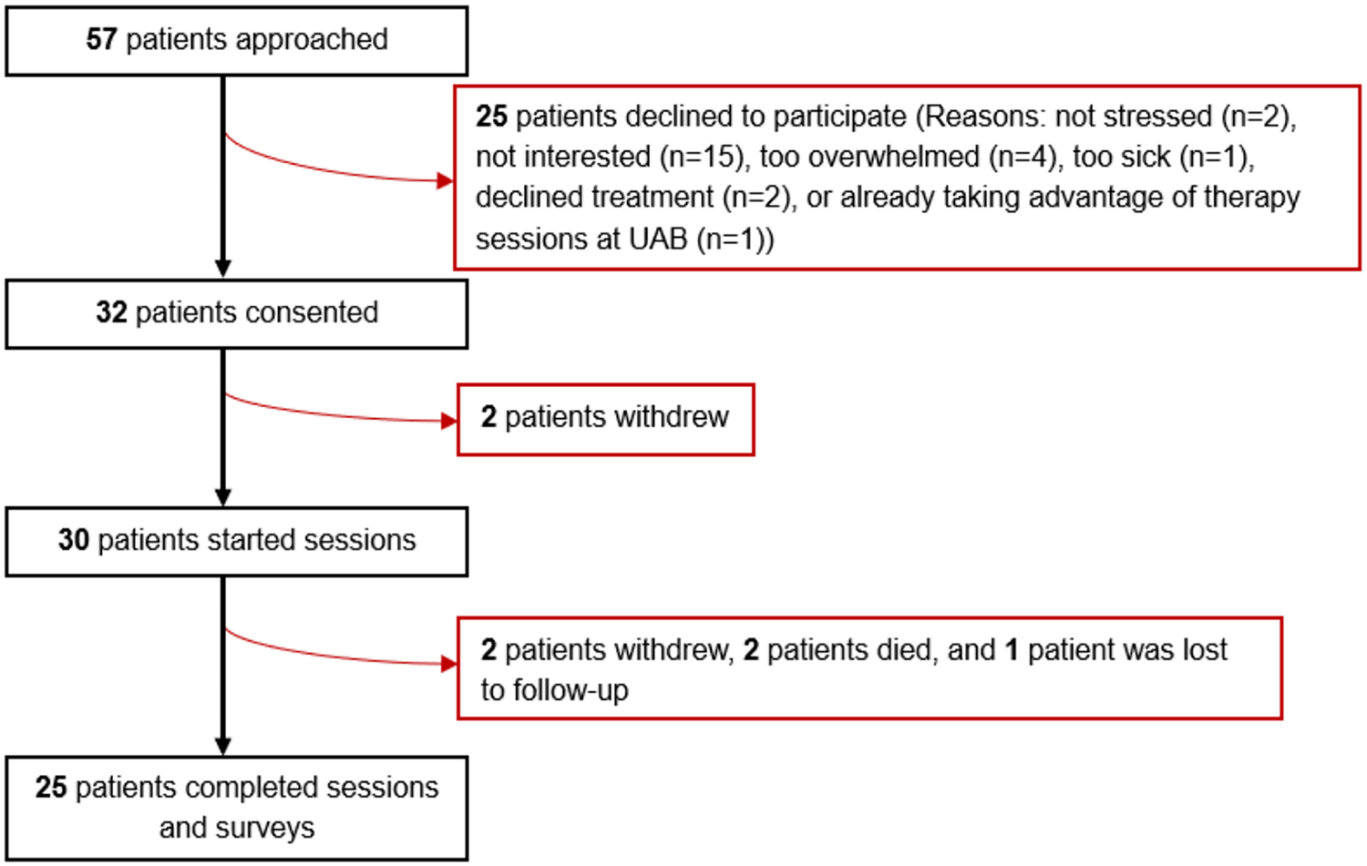
Consort diagram

Of those initiating PRISM, two died during the intervention period, two withdrew (after sessions 2 and 3), and one was lost to follow-up (Fig. 1). Thus, 25 participants (83%) who initiated the intervention completed all 6 coaching sessions and pre-post surveys, surpassing our primary feasibility endpoint of 70%. Two participants completed 1 session each in person, and two participants did all sessions by telephone. The remaining used video. Additionally, participants reported that the sessions met secondary survey-based implementation outcomes of feasibility [FIM mean 4.6 (SD 0.4)], acceptability [AIM mean 4.6 (SD 0.7)], and appropriateness [IAM mean 4.5 (SD 0.9)]. Of the 30 participants, 13 downloaded the app.

### Quantitative exploratory outcomes

**Fig. 2:**
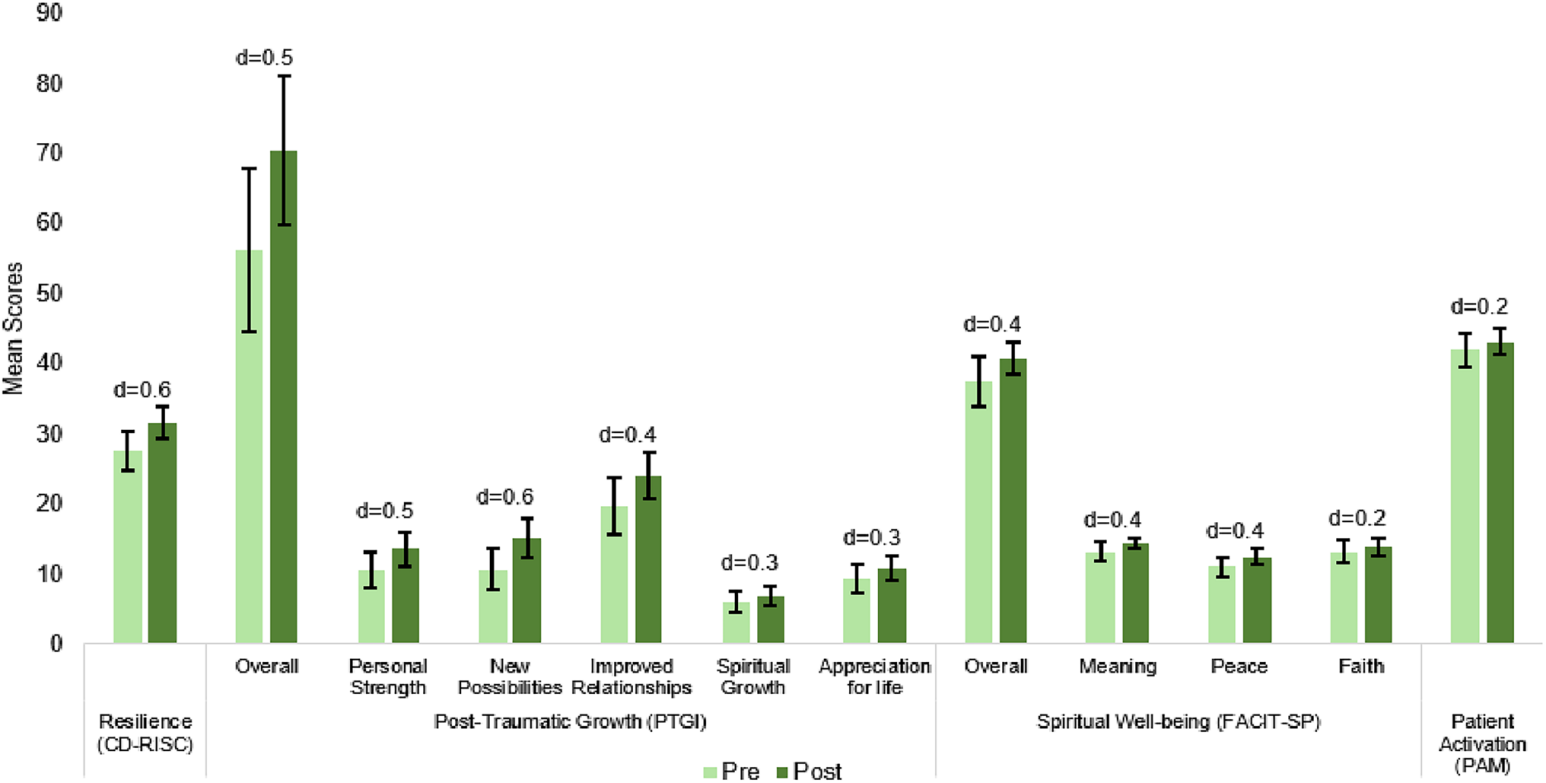
Pre- and post-PRISM intervention scores for secondary outcomes where higher scores are favorable (*N* = 25); Error bars represent confidence intervals

**Fig. 3:**
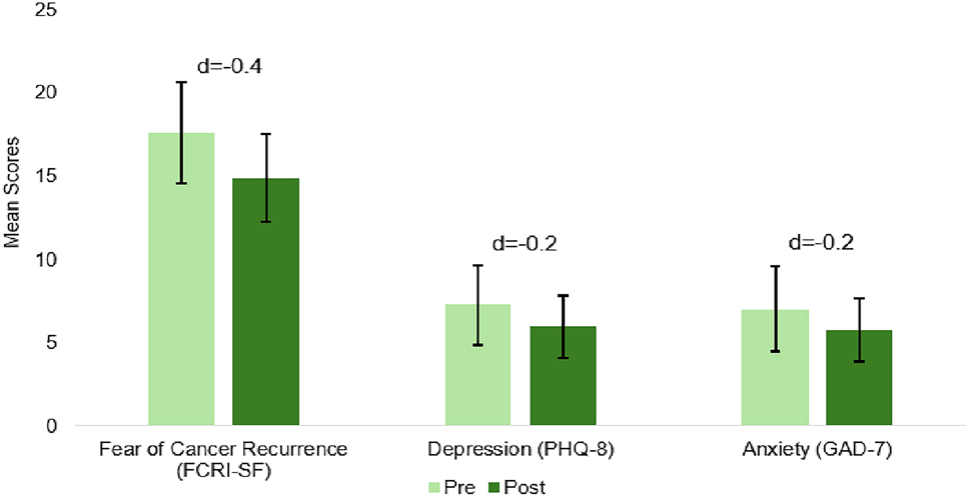
Pre- and post-PRISM intervention scores for secondary outcomes where lower scores are favorable (*N* = 25); Error bars represent confidence intervals

**Fig. 4:**
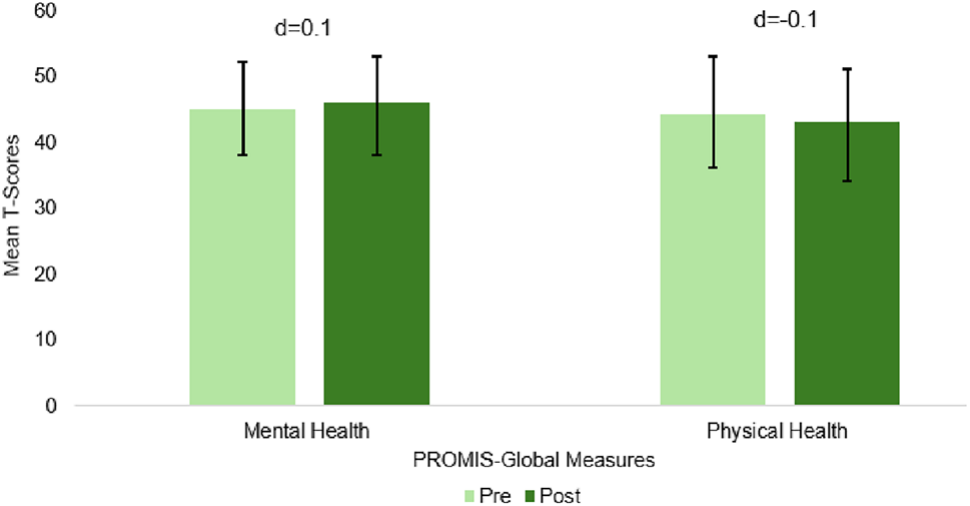
Pre- and post-PRISM intervention scores for secondary outcomes: PROMIS-Global Mental and Physical Health (*N* = 25); Error bars represent confidence intervals

The largest pre-post change for the PRISM intervention was observed in the Post Traumatic Growth Inventory (PTGI: + 14-points; *d* = 0.5; Fig. 2). In a subscale analysis for post-traumatic growth, the greatest areas of change were within “New Possibilities” (+ 4.5-points, *d* = 0.6) and “Improved Relationships” (+ 4.2-points, *d* = 0.4). Other factors, including Personal Strength, Appreciation for Life, and Spiritual Growth, also showed improvements (+ 3-points, *d* = 0.5; + 1.5-points, *d* = 0.3; + 1-point, *d* = 0.3, respectively). Significant pre-post changes were also observed in participant-reported resilience by the Connor Davidson scale (CD-RISC: + 4-points, *d* = 0.6). Additionally, participants showed meaningful improvements in overall spiritual well-being (FACIT-Sp: + 3-points, *d* = 0.4) with the most significant increase in peace (+ 1.4-points, *d* = 0.4), meaning (+ 1.3-points, *d* = 0.4), and faith (+ 1-point, *d* = 0.2). Patient activation also rose modestly pre- to post-PRISM (PAM: + 1.2-points, *d* = 0.2; Fig. 2). In addition to improvements in positive psychological measures, participants also experienced reductions in fear of cancer recurrence pre- vs. post-PRISM intervention (FCRI-SF: − 2.7-points, *d* = − 0.4), as well as in depression (PHQ-8: − 1.3-points, *d* = − 0.2), and anxiety (GAD-7: − 1.2-points, *d* = − 0.2; Fig. 3). Over half (52%) of participants experience anxiety and find everyday activities somewhat difficult post-PRISM interventions, but 7% less participants reported having trouble with everyday activities, and 23% more participants reported no difficulty at all (GAD-7). However, overall mental health showed a slight improvement (PROMIS-Global: + 1-point, *d* = 0.1; Fig. 4), despite participants reporting a decline in physical health, both in the self-assessed question (PROMIS-Global: − 0.2-points, *d* = − 0.2) and summed question (PROMIS-Global: −1-point, *d* = − 0.1).

### Qualitative perceptions of the PRISM intervention

The interview data mirrored findings from the survey data. Participants perceived the PRISM intervention as “very useful and something to put into practice,” as expressed by one participant. They discussed how PRISM enabled post-traumatic growth by encouraging participation in new possibilities, despite their cancer diagnosis. Another participant stated, through PRISM “people need to see that… diagnosis [does not equate the] mentality that ‘my life is over’…I was living, I was thriving…I went to New Orleans…on a retreat…a cruise…to the beach.” The experience encouraged her to seize opportunities as they arose and to live her life.

This growth also translated into improved relationships, as noted by one participant who expressed changes in her comfort with sharing emotions with others. She stated that prior to PRISM, “I had tried to deal with depression…on my own because I do not like to bother anyone with what’s going on with me…I have gotten a little better at…[sharing with others] since I have been doing the PRISM.” These positive changes were echoed by another participant who described how PRISM bolstered her resilience and helped her “to just mentally walk my way through my stress and to try to find a solution and to set out goals.” Another participant perceived PRISM as “something to help me emotionally and to be more, to keep my sanity and my compassion and my resilience up.” They also highlighted how specific programmatic aspects were integrated into their daily life, as reported by a participant who shared “every morning…I open with prayer and then I talk to her [PRISM coach] and we do these little breathing exercises.”

These tools were also influential in combating negative psychological outcomes. One participant stated she had “felt anxious of being around other people because [of] anything that could delay my treatment. I used my PRISM app and I started doing the deep breathing techniques. However, I went in into how to switch the thoughts. I type it in the app and I follow the steps they give me.” Additionally, in regard to treatment-associated anxiety, she mentioned, “I just have to visualize positive things [in relevance to PRISM session on visualization] and trying to get myself away from the MRI. It works.”

Finally, some participants contextualized how PRISM provided knowledge and confidence about how to become more active in terms of their healthcare. For example, a participant expressed that “[she] took some of the training [PRISM tools] that I learned with me like when I go to the doctor with me while I'm waiting on them to come in.” Ultimately, patients found the PRISM intervention to be a positive psychological support tool.

### Mixed methods

The patient perspectives from the interviews both provide supportive evidence not only of feasibility and acceptability but also suggest potential mechanisms for the changes observed in the patient-reported outcomes. There were direct and specific references to key scenarios demonstrating how the intervention supported resilience and positive psychology as found in the survey data, coupled with specific examples of how participants used the intervention to mitigate negative psychologic experiences.

## Discussion

This pilot study demonstrates the preliminary feasibility of applying the PRISM intervention for women with early stage breast cancer who are receiving chemotherapy. Feasibility was further supported by patient reports of acceptability, feasibility, and appropriateness from validated surveys, patient interviews, as well as encouraging pre-post changes in key psychological outcomes. This study extends the application of PRISM to a large, novel population of more than 300,000 women diagnosed with early stage breast cancer annually in the United States [36] with substantial opportunity for intervention. Given the high prevalence and incidence of breast cancer and increases in treatment options, there are increasing numbers of women in survivorship facing adverse psychological outcomes who have potential to maximize quality of life via resilience interventions.

While this pilot was not designed for formal outcomes testing, the exploratory outcome data was directionally consistent with anticipated PRISM benefits and similar to prior studies of PRISM in other populations. For example, among AYAs receiving chemotherapy for cancer, PRISM improved participant-reported resilience, hope, and quality of life, while reducing psychological distress [12, 13], and improvements were durable 2 years later [20]. Among AYAs who reported psychological distress while receiving hematopoietic cell transplantation for hematologic malignancies, PRISM improved hope and symptoms of anxiety [16]. Likewise, among distressed AYAs with Type 1 Diabetes, PRISM improved mental health outcomes [15]. In all studies, PRISM’s impacts were more pronounced in its direct targets of positive psychology domains, including hope, resilience, and quality of life. These results align with our study, which demonstrated the greatest impact in resilience and post-traumatic growth, which are key targets of PRISM. In addition, key benefits were observed in all associated domains of post-traumatic growth, patient activation, fear of recurrence, spiritual well-being, depression, anxiety symptoms, global health, and mental health. The only domain in which a decline was observed was physical health, which was expected due to receipt of chemotherapy and would not be expected to be substantially improved through psychosocial intervention. Use of a control group in a future PRISM randomized trial in this population will provide greater insight on treatment effects during the physically taxing period of active treatment. For instance, it is possible that a control group would have experienced even greater decreases in physical health over the study period, with PRISM providing a buffering effect and thus a smaller decline.

Of note, this feasibility study was conducted in a diverse population with a high burden of social needs, with the population being predominantly Black (57%) and having high social needs with 73% reporting trouble paying for basic necessities of food, medical care, housing, or utilities. These patient groups may have prior traumatic experiences and/or concurrent stressors in addition to their cancer diagnosis, such as experiences of systemic racism, persistent poverty, or housing instability, which could influence their cancer experience and outcomes. The demonstrated feasibility with high levels of activation in this at-risk population, coupled with existing data on the relationship between breast cancer and stress, suggests that PRISM may be uniquely beneficial in this population.

It is notable that the largest effect sizes from pre- to post-intervention for this PRISM-EBC study were observed for resilience and post-traumatic growth, both positive psychological outcomes. This contrasts with the smaller effects on reductions in the negative outcomes of depression and anxiety. This pattern may stem from the nature of the intervention, which focuses on building positive coping skills and positive psychology as a means to address cancer-related distress. Additionally, because this intervention primarily focuses on building resilience rather than treating mood/anxiety symptoms, there was no distress minimum for inclusion in this study. As such, the smaller effect sizes for negative outcomes of anxiety and depression may be due to mild levels of symptomatology present at baseline (floor effects). Finally, it is possible that positive psychology constructs may serve a mechanistic function, by buffering” the effects of stressors on negative psychological states. Future larger trials could include additional follow-up time points and exploration of mediational models for the intervention.

A strength of this intervention is the remote nature with telehealth coaching calls and a companion app, increasing implementation and dissemination feasibility. This study adds to a growing literature on telehealth and digital intervention programs demonstrating psychological and physical well-being benefits in adult patients with cancer [37, 38]. Little work has been done to examine feasibility and acceptability of such technology-based interventions in marginalized breast cancer populations (i.e., racial/ethnic minority and low SES), a crucial contribution of this study. Additionally, the majority of interventions in breast cancer have focused on alleviating distress, measuring negative psychological and physical health outcomes, rather than enhancing resilience and assessing positive psychology constructs, a unique aspect of this work. Positive psychological factors have been associated with positive health behaviors and improved health outcomes in people with serious illnesses, including cancer [39–43], highlighting the value of an intervention that can successfully target enhancement of such protective factors and may impact outcomes independent of distress. Positive psychology interventions, while more limited in number, also tend to be well-accepted and cost-effective, increasing likelihood of widespread uptake [44, 45]. Furthermore, this study provides a first step in applying this previously successful intervention, initially designed for an AYA population, more broadly for adults with cancer. If the next step of this work confirms efficacy of PRISM in breast cancer, this program may also have utility in other cancer types and diseases.

This study has several limitations. As this was an initial feasibility study, the sample size was small, there was no control comparison, and outcomes focused on feasibility. Thus, our study was not designed to evaluate efficacy, although such evaluation is planned in a subsequent trial. Sample heterogeneity regarding cancer subtype and time since initiation of treatment could influence participants’ psychological experiences while receiving PRISM intervention, and the sample size of this study did not permit sub-group analyses to address these differences. This study was also conducted at a single site and findings may vary in geographically or demographically different samples. More research is needed to understand if virtually delivered vs. in-person interventions can yield similar psychobiological processes and impact longer-term health outcomes [46–49]. In addition, further assessment is needed to assess impact within different subtypes (e.g. triple negative, estrogen receptor positive) and based on different treatments received (e.g. anthracyclines). Thus, future trials of PRISM in breast cancer will be needed to expand outcome assessments to include psychophysiological indicators and stress-related biomarkers (e.g., heart-rate variability, allostatic load), as well as cancer clinical outcomes.

## Conclusion

The PRISM intervention was feasible among a diverse group of women with early stage breast cancer, and effect sizes suggest potential benefit in psychological outcomes, warranting further investigation.

## Data Availability

All feasibility and psychosocial data collected and analyzed during the study are included in this manuscript. De-identified data may be available with IRB approval upon request.
